# Inequality in Social Support Associated With Mild Cognitive Impairment: A Cross-Sectional Study of Older (≥60 Years) Residents in Shanghai, China

**DOI:** 10.3389/fpubh.2021.706322

**Published:** 2021-11-23

**Authors:** Yuan Lu, Chaojie Liu, Sally Fawkes, Jia Ma, Yalin Liu, Dehua Yu

**Affiliations:** ^1^Department of General Practice, Yangpu Hospital, Tongji University School of Medicine, Shanghai, China; ^2^School of Psychology and Public Health, La Trobe University, Melbourne, VIC, Australia; ^3^Community Health Service Centre of Dinghai in YangPu District, Shanghai, China; ^4^Community Health Service Centre of JiaDing Town in JiaDing District, Shanghai, China; ^5^Shanghai General Practice and Community Health Development Research Center, Shanghai, China

**Keywords:** social support, mild cognitive impairment, socioeconomic, inequality, income, cross-sectional, China

## Abstract

**Objective:** Social support plays a critical role in the detection and management of mild cognitive impairment (MCI). However, socioeconomic inequalities exist in both social support and health care services. Our study aimed to compare the level of social support received by MCI patients in comparison with those without MCI and to determine its link with income.

**Methods:** Secondary data analyses were performed. Social support was measured using the Duke Social Support Index (DSSI) and satisfaction ratings. Multivariate logistic regression models were constructed to determine the associations of personal income and MCI with social support after adjustment for variations in the sociodemographic and health characteristics of the respondents. The multiplicative and additive interaction effects of income and MCI were further examined through introducing the MCI^*^Income variable to the regression models and using the relative excess risk due to interaction (RERI) analysis, respectively.

**Results:** The logistic regression models showed that the respondents with MCI had significantly lower social support as measured by the DSSI scores (AOR = 33.03, *p* < 0.001) and satisfaction ratings (AOR = 7.48, *p* < 0.001) compared with those without MCI. Similarly, social support decreased with lower personal income (*p* < 0.001). There existed a significant multiplicative interaction effect between personal income and MCI on social support (AOR = 0.30–0.32, *p* < 0.01). The gap in social support between those with and without MCI was higher in the higher income group compared with the lower income group (*p* < 0.001). No significant additive interaction effects on social support were found between MCI and income.

**Conclusions:** There are significant disparities in social support between people living with and without MCI. Such a gap is more profound in people with higher income. The inequality in social support associated with MCI may present a significant challenge to the successful implementation of community MCI detection and management.

## Introduction

The prevalence of dementia is projected to double in 20 years worldwide and in 10 years in China (1). It is predicted that about 7% of Chinese people over 60 years old (23.3 million) will experience dementia by the year of 2030 (2, 3). The disability and care burden resulting from dementia imposes serious financial burdens on health care systems and societies (2, 3). Accordingly, dementia has been established as a global health priority by the World Health Organisation (4). Due to a prevailing lack of effective treatment regimens, the most cost-effective measure for managing dementia is to intervene at an early stage to slow its progress (5). Mild cognitive impairment (MCI) is deemed an intermediate state between normal cognitive ageing and dementia (6). Previous studies indicated that the prevalence of dementia would be halved if successful MCI interventions could delay the onset of dementia by 5 years (7). Therefore, early detection and effective management of MCI are critical.

There are effective MCI intervention measures available to slow the progress of cognitive decline. A systematic review (8) found moderate level evidence to support the effectiveness of cognitive intervention measures to delay the progress of MCI towards dementia, although the efficacy of pharmacological interventions has not yet been confirmed (9). These include cognitive stimulation (10), cognitive training (11), and cognitive rehabilitation (12), as well as management of some chronic conditions that are associated with MCI and its progression to dementia (13). However, implementation of these intervention measures can be challenging due to the high frequency and intensity of engagement required (11). Patient cooperation and compliance depend on high levels of engagement of affected individuals and a supportive social environment. Many people are not aware of the need for medical attention for mild memory problems, and are reluctant to seek help. Previous studies indicate that about two-thirds of people with dementia and over 90% of people with early dementia were not noted in the primary care setting in the United States (14). Based on the Chronic Care Model (15), one cause of ineffective management of chronic illnesses may be a mismatch between the attitudes of practice teams and patients, as well as a lack of a system environment that enables effective interactions between the two. There exists poor understanding in the general public about MCI and dementia. It is not uncommon for people to consider MCI as part of normal ageing that does not warrant treatment (16). Cognitive decline is often stigmatised in societies. Diagnoses and disclosure of MCI may be embarrassing from the perspectives of health workers, patients and their families (17). People may pretend that this problem would not affect themselves and their loved ones. MCI services are usually invisible, if they ever even exist, in communities (18).

Social support plays a critical role in the detection and management of MCI. Social support refers to the process through which social relationships promote health and well-being (19). Empirical evidence shows that individuals with high levels of social support are more likely to pursue healthy enhancing personal habits and are more willing to confront health problems and seek medical care when needed (20). Poor social relationships have been shown to be associated with MCI (21). Social support gives people the experience of being loved, cared for, respected, and belonging to a network of communication. This can serve as a powerful tool for people to overcome stigma attached to MCI and seek needed healthcare services (22). Social support is particularly important for MCI management given that it is usually accompanied by reduced participation in social activities as a result of cognitive difficulties (23). Social support can offer opportunities for early detection of cognitive and behavioural changes in people (24). Furthermore, an extensive social network can help MCI patients to get access to social resources, establish self-esteem, and reduce self-isolating behaviours (25).

Unfortunately, socioeconomic inequalities exist in both social support and health care services (26). People living in socioeconomically disadvantaged communities tend to have lower levels of social support and are likely to suffer from more health risks compared with their counterparts with high socioeconomic status (27). Certain resources are usually required to participate in social activities (28). Empirical evidence shows that people living with higher income usually have more resources and better access to social support than the poorer ones (27). Poverty can even lead to social exclusion (27). Although the risk of developing MCI is almost double that in people living in the lowest quartile of socioeconomic status compared to the highest quartile, according to a seven-year cohort study (29), low socioeconomic status may jeopardise the chance of the MCI patients with low income obtaining sufficient social support.

This study aimed to compare the level of social support received by MCI patients with those without MCI and to determine its link with personal income. Our current understanding of the interaction effect between income and MCI on social support is quite limited (30). This study addresses this gap in the literature. The study was conducted in Shanghai, China. Over the past few decades, there has been unprecedented economic growth and rapid ageing in populations in China (31). However, there has been significant concern expressed about increased disparities in social, economic and health development. Such disparities are particularly profound in rapidly growing urban areas (27). In low and middle income countries, including China, people of low socioeconomic status are exposed to environments that produce behavioural health risks (26). Socioeconomic disadvantage is also a risk factor for cognitive decline and other chronic non-communicable diseases (32, 33). However, it would be naïve to expect increased income to offer a solution to the challenges of high behavioural risks and low social support that MCI patients are confronting. Previous studies indicate that cognitive disorders are associated with poor social support in older adults (25, 34). It is unclear to what extent increased income could help mitigate the risk of declined social support in MCI patients. This study will advance our understanding of this issue by testing the following hypotheses using an existing cross-sectional survey dataset:

1. MCI is associated with low social support compared with those without MCI;
2. MCI interacts with income in its association with social support.

## Methods

Secondary data analyses were performed using the data collected through a cross-sectional survey of residents aged 60 years or older in Shanghai, China. Ethics approval was obtained from the Human Ethics Committee of La Trobe University (HEC20125).

### Study Setting

The study was conducted in Shanghai, one of the most developed cities in China. Shanghai is the first city that has surpassed the benchmark of an ageing society in China and experienced a negative population growth (35). About 5.18 million (35.2%) of its residential population were older than 60 years in 2019 (36). Shanghai had a GDP per capita of ¥134,982 Yuan (US$20,766) in 2019, ranking top two among the 31 regions in mainland China (37).

### Data Source

Data were drawn from a cross-sectional survey on MCI. The survey was organised by the Shanghai MCI study alliance over the period from June 2018 to May 2019, with an aim to prepare for community management of MCI. The survey followed a procedure in line with the World Medical Association Declaration of Helsinki, which was approved by the two coordinating community health centres (CHCs) of the MCI study alliance (LS2018-1 and JD201801). Informed consent was obtained from each participant prior to the survey. Access to the de-identified data for the purpose of this study was granted by the Shanghai MCI study alliance. Extensive use of the data for research purposes is encouraged by the alliance.

Community residents aged 60 years or over from the geographic catchments covered by the two CHCs in Shanghai (Jiading town and Dinghai) were eligible to participate in the survey. Jiading town is located in the west of Shanghai with a registered population of 16,991 over 60 years old, while Dinghai is located in the east of Shanghai with a registered population of 33,731 over 60.

**Figure 1:**
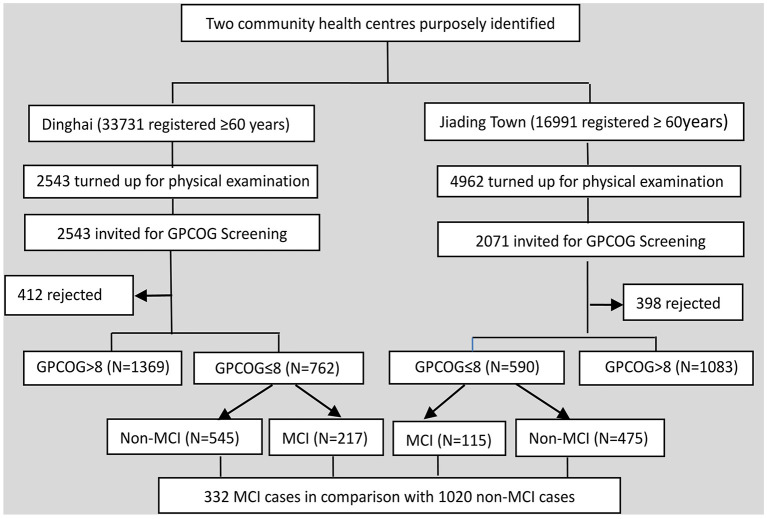
Study flow diagram. GPCOG, General Practitioner Assessment of Cognition-Chinese version; MCI, mild cognitive impairment.

The survey participants were recruited from those who attended the annual free physical examinations offered to older adults (≥60 years) by the two CHCs. About 15% of the registered residents over 60 years received the free physical examinations over the study period. In Dinghai, all of the 2,543 attendees were invited to participate in the study, compared with a convenience sample of 2071 (41.7%) out of the 4,962 attendees in Jiading town (Figure 1).

### Data Collection

#### Mild Cognitive Impairment (MCI)

A two-phase protocol was adopted to identify MCI in the study participants. In the first phase, the General Practitioner Assessment of Cognition-Chinese version (GPCOG-C) (38) was used as a screening tool. Those who had a score equal to or under 8 (38) were referred to a neurologist for clinical diagnosis of MCI in phase two. The diagnosis of MCI followed the Petersen criteria: (1) memory complaints; (2) memory impairment assessed by the Montreal Cognitive Assessment (MoCA) tool (39); (3) intact activities of daily living (ADL) (40); and (4) absence of dementia according to the clinical dementia rating (CDR) (41).

#### Social Support

Social support was defined as the social resources provided by non-professionals either from formal support groups or informal helping relationships (19). According to Cohen et al. (42), social support can be examined through an objective lens (e.g., volume and strength) or subjective experiences (e.g., loneliness). The Duke Social Support Index (DSSI) (43) instrument was adopted to assess social support. It contains three domains: Instrumental Social Support (ISS, 12 items) measuring help with tangible needs; Social Interaction Scale (SIS, 4 items) measuring an emotional relationship with others, and Subjective Social Support (SSS, 7 items) measuring subjective perceptions of being respected, supported and understood. The DSSI instrument has been widely used in various studies (43) and validated in people living with MCI and in China (44–46). In this study, an additional item was added to ask respondents to rate their overall satisfaction with social support received.

#### Sociodemographic Characteristics

The survey gathered data regarding the sociodemographic characteristics of the study participants, their health and health behaviours. Many factors can influence the availability and strength of social support. Dunkel-Schetter and Skokan (47) classified these factors into four aspects. They suggest that the likelihood of a person seeking support from others often depends on the felt urgency, availability and accessibility of support, and the perceived impacts of accessible support. These four factors correspond well with the three predictors of healthcare seeking behaviours in Andersen's model: need (perceived impacts), predisposing (felt urgency), and enabling (availability and accessibility) (48). In this study, comorbidity (chronic conditions) and health behaviours (smoking, drinking alcohol, not having regular exercises) were considered to be a major set of factors that could influence the need for people to seek support. Respondents were asked to report whether they had any chronic diseases (such as hypertension, diabetes, stroke, heart diseases, cancer, chronic respiratory diseases, and others) that had been diagnosed by a medical doctor. Drinking alcohol was defined as consuming alcohol more than once per month over the past six months (49). A person was defined as ‘smoking' if they were currently smoking and had consumed at least 100 cigarettes in the past (50). Regular exercise was defined as having three or more occasions of at least 30-min of moderate physical activity per week (51).

Age and gender were deemed predisposing factors in this study. Past studies found that older adults tend to have smaller social networks; yet age has a U-shaped relationship with social support (52, 53). A meta-analysis concluded that women give and receive higher social support than men (54). Women also appear to benefit slightly more from high social support than men (55).

Enabling factors measured in this study included income, educational attainment, occupation, marriage, having children, and co-habiting with others. Income is not only important for enabling social support, but is also assumed in this study to moderate the association between MCI and social support. Education can shape help seeking behaviours, including acquisition of social support (56).

People spend about 10% of their lifetimes in working environments (57), which could have a profound impact on the availability and accessibility of social support. A married person is likely to be encouraged by their partner to seek help (58). Similarly, those who have children and co-habit with others are also likely to be encouraged by their children and housemates to seek help.

The survey was completed by trained health workers through face-to-face interviews. On average, the entire screening and survey process took about 40 min.

### Measures

#### Dependent Variable

The level of social support was the primary outcome indicator (dependent variable) in this study. Respondents were asked to answer “1 = Yes” or “0 = No” to each ISS item. The SIS items were rated on a three-point scale ranging from 1 (few) to 3 (many), while the SSS items were rated on a three-point scale ranging from 1 (little) to 3 (much). A summed score was calculated for the entire DSSI instrument (ranging from 11 to 45) and its three domains (0–12 for ISS; 4–12 for SIS; 7–21 for SSS), with a higher score indicating higher social support. In this study, social support was categorised into two levels, with the middle point (28) of the DSSI score range serving as a cut-off point. Similar approaches have been widely applied in the literature (59). Unlike mean-split or median-split, it has the advantage of objectiveness, being independent of the sample data.

Self-rating of overall experience on (satisfaction with) social support was assessed using a six-point Likert scale ranging from 1 (less than what is needed) to 6 (more than what is needed), which was subsequently categorised into two levels: low (<3 “with unmet needs”) and high (≥3 “without unmet needs”).

#### Independent Variable

The association of income and MCI with social support, including their interaction effect, was the major interest of this study. This study assessed individual disposable income by asking respondents how much money they had available that they could spend every month from multiple sources. We divided respondents into high (≥¥4000) and low (< ¥4000) income groups. The cut-off point was chosen based on the average monthly income for retired people, similar to the mean income reported by the study participants, but lower than the average disposable income in Shanghai (37).

MCI was diagnosed through a two-phase process as described in the data collection section.

#### Covariates

Covariates were coded as categorical variables, including age (60–69, 70–79, ≥80 years), gender (male, female), marriage (yes, no), educational attainment (primary, middle, high or above), children (yes, no), co-habiting arrangements (yes, no), occupation (office, manual), satisfaction with disposable income (yes, no), chronic conditions (yes, no), smoking (yes, no), drinking alcohol (yes, no), and regular exercise (yes, no). We classified occupation into office work (administration officer, doctor, teacher, engineer, artist, banking executive) and manual work (agricultural work, factory worker, driver, and sales and services), because they have varied availability and accessibility to social support (60). The health behaviour variables (smoking, drinking, and exercise) were defined and coded in line with the existing literature (49–51).

### Statistical Analysis

The characteristics (categorical variables) of study participants were described through frequency distributions and compared between the two participating CHCs using Chi-square (χ^2^) tests. Means and standard deviations (SD) of reported disposable income were calculated and compared between the two CHCs using student *t*-tests.

The summed DSSI scores (including the ISS, SIS and SSS scores) were described using median and inter-quartile range (IQR) values and compared between the respondents with different characteristics through Mann-Whitney (two-group) tests or Kruskal-Wallis (multiple-group) tests. The level of social support (including both DSSI and self-ratings) was further categorised into two groups (low vs. high) and compared between those with and without MCI using χ^2^ tests.

Multivariate logistic regression models were established with an enter approach (all independent variables and covariates were entered in a single step as a block) to determine the associations of income and MCI with the social support indicators after adjustment for variations in other variables. The multiplicative interaction effect of personal income and MCI was further examined by introducing the interaction term “income^*^MCI” into the logistic models. The unadjusted and adjusted odds ratios (OR) and 95% confidence intervals of the predictors were presented.

The additive interaction effect between MCI and personal income was tested using three indicators: the relative excess risk due to interaction (RERI); the attributable proportion due to interaction (AP); and the synergy index (S). Calculation of the three indicators followed the delta method using the Excel sheet developed by Knol et al. (61). There is no additive interaction if RERI and AP equal to 0, and S equals to 1.

To test the robustness of the findings, logistic regression modelling was also performed for the DSSI scores categorised by the median value. The tests produced consistent results (Supplementary Table 1).

The analyses were performed using SPSS software version 27.0. A two-sided *p* value < 0.05 was considered as statistically significant.

## Results

### Characteristics of Participants

The study participants had a mean age of 70.4 (SD = 6.8) years: 48.7% were younger than 70 years. Women accounted for 65.8% of the study participants. The majority of study participants was married (90.5%), had children (95.5%), co-habited with others (81.7%), completed middle or higher school education (71.7%), and engaged in manual labour work (56.8%). About half of respondents (51.8%) reported chronic conditions; 13.3% and 15.5% were currently smoking and drinking alcohol, respectively; and 64.4% were regularly exercising.

Compared with those in Dinghai, the study participants from Jiading town were younger, and less likely to be married, to have children, to co-habit with others and were regularly exercising, but more likely to engage in office work and be drinking alcohol and smoking. The participants from Jiading town reported lower disposable income (3,778.30 ± 752.57) than those from Dinghai (4,313.78 ± 1,123.81, *p* < 0.001).

In total, 24.6% of the study participants were diagnosed with MCI: 28.5% in Dinghai compared with 19.5% (*p* < 0.001) in Jiading town. Higher levels of social support were found in the study participants in Jiading town compared with those in Dinghai (*p* < 0.05) (Table 1).

**Table 1:** Demographic characteristics of the participants (*n* = 1,352).

**Characteristics**	**Number and Percentage (%) of Respondents**			***p***
	**Dinghai (*n* = 762)**	**Jiading (*n* = 590)**	**Total (*n* = 1,352)**	
**Age (years)**				0.001
60–69	338 (44.4%)	320 (54.2%)	658 (48.7%)	
70–79	394 (51.7%)	248 (42.1%)	642 (47.5%)	
≥80	30 (3.9%)	22 (3.7%)	52 (3.8%)	
**Gender**				0.862
Male	259 (34.0%)	204 (34.6%)	463 (34.2%)	
Female	503 (66.0%)	386 (65.4%)	889 (65.8%)	
**Marital status**				0.001
Yes	708 (92.9%)	516 (87.5%)	1,224 (90.5%)	
No	54 (7.1%)	74 (12.5%)	128 (9.5%)	
**Educational level**				0.224
Primary school	203 (26.6%)	180 (30.5%)	383 (28.3%)	
Middle school	502 (65.9%)	374 (63.4%)	876 (64.8%)	
High school and above	57 (7.5%)	36 (6.1%)	93 (6.9%)	
**Occupation**				<0.001
Office work	269 (35.3%)	315 (53.4%)	584 (43.2%)	
Manual Labour work	493 (64.7%)	275 (46.6%)	768 (56.8%)	
**Living arrangement**				<0.001
Alone	112 (14.7%)	136 (23.1%)	248 (18.3%)	
Cohabiting with others	650 (85.3%)	454 (76.9%)	1,104 (81.7%)	
**Having children**				<0.001
Yes	761 (99.9%)	530 (89.8%)	1,291 (95.5%)	
No	1 (0.1%)	60 (10.2%)	61 (4.5%)	
**Comorbidity**				0.100
Yes	380 (49.9%)	321 (54.4%)	701 (51.8%)	
No	382 (50.1%)	269 (45.6%)	651 (48.2%)	
**Currently drinking**				<0.001
Yes	59 (7.7%)	151 (25.6%)	210 (15.5%)	
No	703 (92.3%)	439 (74.4%)	1,142 (84.5%)	
**Currently smoking**				0.002
Yes	82 (10.8%)	98 (16.6%)	180 (13.3%)	
No	680 (89.2%)	492 (83.4%)	1,172 (86.7%)	
**Regular exercise**				<0.001
Yes	549 (72.0%)	322 (54.6%)	871 (64.4%)	
No	213 (28.0%)	268 (45.4%)	481 (35.6%)	
**Mild cognitive impairment**				<0.001
Yes	217 (28.5%)	115 (19.5%)	332 (24.6%)	
No	545 (71.5%)	475 (80.5%)	1,020 (75.4%)	
**Personal disposable income (Yuan)**				
Low (<4,000)	267 (35.0%)	353 (59.8%)	620 (45.9%)	<0.001
High (≥4,000)	495 (65.0%)	237 (40.2%)	732 (54.1%)	
**Satisfaction with income**			
Low	310 (40.7%)	268 (45.4%)	578 (42.8%)	0.086
High	452 (59.3%)	322 (54.6%)	774 (57.2%)	
**Social support score**				
Low (<28)	359 (47.1%)	203 (34.4%)	562 (41.6%)	<0.001
High (≥28)	403 (52.9%)	387 (65.6%)	790 (58.4%)	
**Self-ratings on social support**				
Low (<3)	110 (14.4%)	61 (10.3%)	171 (12.6%)	0.026
High (≥3)	652 (85.6%)	529 (89.7%)	1,181 (87.4%)	

### Social Support

The respondents had a median DSSI score of 29 (IQR 18–31). Those without MCI (30, IQR 21–31) had significantly higher DSSI scores than those with MCI (16, IQR 14–19, *p* < 0.01). Similar results were found in the ISS, SIS and SSS domain scores. More than 72% of the respondents without MCI were classified as having high social support (DSSI score ≥ 28), compared with 16.3% in those with MCI. The gap in satisfaction with social support between the respondents with and without MCI was smaller (91.5% vs. 74.7%), but were still statistically significant (*p* < 0.001) (Table 2).

**Table 2:** Social support for the study participants with and without MCI (*n* = 1,352).

**Social support**	**Total**	**Respondents without MCI (*n* = 1,020)**	**Respondents with MCI (*n* = 332)**	***p***
**Social support score [Median (Inter-Quartile Range)]**				
Duke Social Support Index (DSSI)	29 (18–31)	30 (21–31)	16 (14–19)	0.005
Instrumental Social Support (ISS)	7 (5–8)	8 (6–9)	4 (2–5)	<0.001
Social Interaction Scale (SIS)	7 (5–8)	8 (6–8)	4 (4–6)	<0.001
Subjective Social Support (SSS)	13 (7–16)	14 (10–17)	7 (7–9)	<0.001
**DSSI category [n (%)]**				<0.001
Low (<28)	562 (41.6%)	284 (27.8%)	278 (83.7%)	
High (≥28)	790 (58.4%)	736 (72.2%)	54 (16.3%)	
**Self-rating on social support [n (%)]**				<0.001
Low (<3)	171 (12.6%)	87 (8.5%)	84 (25.3%)	
High (≥3)	1,181 (87.4%)	933 (91.5%)	248 (74.7%)	

### Socio-Demographic Factors Associated With Social Support

Lower social support, as indicated by all of the three indicators (DSSI score, DSSI category, satisfaction with social support), was found in study participants who were older, not married, engaged in manual labour work, reported chronic conditions, and had lower satisfaction with income. Currently drinking alcohol was associated with higher DSSI scores, but not with satisfaction with social support; whereas, regularly exercising was associated with higher satisfaction with social support, but not with the DSSI scores. Women, those who completed up to middle school education, had children, co-habited with others, and had higher disposable income reported higher DSSI scores and had higher levels of social support than others despite there being no difference in self-ratings on social support (Table 3).

**Table 3:** Sociodemographic factors associated with social support.

**Variables**	**DSSI score**			**Level of Social support, n (%)**			**Self-rating on social support, n (%)**		
	**Median**	***IQR***	***p***	**Low**	**High**	***p***	**Low**	**High**	***p***
**Age (Years)**			<0.001			<0.001			<0.002
60–69	30	19–33		196 (29.8%)	462 (70.2%)		65 (9.9%)	593 (90.1%)	
70–79	21	16–30		329 (51.2%)	313 (48.8%)		94 (14.6%)	548 (85.4%)	
≥80	18	15–29		37 (71.2%)	15 (28.8%)		12 (23.1%)	40 (76.9%)	
**Gender**			0.021			**0.017**			0.797
Male	28	18–31		213 (46.0%)	250 (54.0%)		60 (13.0%)	403 (87.0%)	
Female	30	18–31		349 (39.3%)	540 (60.7%)		111 (12.5%)	778 (87.5%)	
**Marital status**			<0.001			<0.001			<0.001
Yes	30	18–31		470 (38.4%)	754 (61.6%)		139 (11.4%)	1,085 (88.6%)	
No	17	15–29		92 (71.9%)	36 (28.1%)		32 (25.0%)	96 (75.0%)	
**Educational level**			<0.001			<0.001			0.058
Primary school	21	16–30		196 (51.2%)	187 (48.8%)		56 (14.6%)	327 (86.4%)	
Middle school	30	19–32		316 (36.1%)	560 (63.9%)		98 (11.2%)	778 (88.8%)	
High and above	21	16–30		50 (53.8%)	43 (46.2%)		17 (18.3%)	76 (81.7%)	
**Occupation**			0.003			<0.001			0.002
Office work	28	18–31		210 (36.0%)	374 (64.0%)		55 (9.4%)	529 (90.6%)	
Manual Labour work	30	19–31		352 (45.8%)	416 (54.2%)		116 (15.1%)	652 (84.9%)	
**Living arrangement**			<0.001			0.008			0.751
Alone	28	16–30		122 (49.2%)	126 (50.8%)		33 (13.3%)	215 (86.7%)	
Cohabit with others	30	18–31		440 (39.9%)	664 (60.1%)		138 (12.5%)	966 (87.5%)	
**Having children**			<0.001			<0.001			0.557
Yes	29	18–31		521 (40.4%)	770 (59.6%)		162 (12.5%)	1,129 (87.5%)	
No	18	14–30		41 (67.2%)	20 (32.8%)		9 (14.8%)	52 (85.2%)	
**Chronic condition**			<0.001			<0.001			0.001
Yes	28	18–31		345 (49.2%)	356 (50.8%)		110 (15.7%)	591 (84.3%)	
No	30	19–32		217 (33.3%)	434 (66.7%)		61 (9.4%)	590 (90.6%)	
**Currently drinking**			0.031			0.128			0.735
Yes	30	19–32		77 (36.7%)	133 (63.3%)		28 (13.3%)	182 (86.7%)	
No	29	18–31		485 (42.5%)	657 (57.5%)		143 (12.5%)	999 (87.5%)	
**Currently smoking**			0.541			0.685			0.905
Yes	30	18–31		72 (40.0%)	108 (60.0%)		23 (12.8%)	157 (87.2%)	
No	29	18–31		490 (41.8%)	682 (58.2%)		148 (12.6%)	1,024 (87.4%)	
**Regular exercise**			0.981			1.000			0.010
Yes	29	18–31		362 (41.6%)	509 (58.4%)		95 (13.3%)	776 (86.7%)	
No	29	18–31		200 (41.6%)	281 (58.4%)		76 (11.9%)	405 (88.1%)	
**Disposable income (Yuan)**			<0.001			<0.001			0.085
Low (<4,000)	28	18–31		291 (46.9%)	329 (53.1%)		89 (14.4%)	531 (85.6%)	
High (≥4,000)	30	18–31		271 (37.0%)	461 (63.0%)		82 (11.2%)	650 (88.8%)	
**Satisfaction with income**			<0.001			<0.001			0.025
Low	16	13–28		318 (55.0%)	260 (45.0%)		87 (15.1%)	491 (85.9%)	
High	30	19–31		244 (31.5%)	530 (68.5%)		84 (10.9%)	690 (89.1%)	

### Interaction Effect Between Income and MCI

The logistic regression models showed that MCI, low personal income, not married, and chronic conditions were factors associated with both low social support (measured by DSSI) and low satisfaction with social support (Table 4). Younger age, women, middle school education, and drinking alcohol were also predictors of high social support measured by DSSI; whereas having children, high satisfaction with income, and a lack of physical exercise were significant predictors of low personal satisfaction with social support. The odds of those with lower personal income receiving low levels of social support (measured by DSSI and satisfaction ratings) were more than twice of those with higher personal income. The multiplicative interaction effect between MCI and income was statistically significant for both DSSI (AOR = 0.30, *p* = 0.001) and satisfaction with social support (AOR = 0.32, *p* = 0.002).

**Table 4:** Logistic regression results of factors associated with social support.

**Predictor**	**DSSI score (1** **=** **Low; 0** **=** **High)**						**Self-rating on social support (1** **=** **Low; 0** **=** **High)**					
	**OR**	**95% CI**	***p***	AOR^*^	**95% CI**	***p***	**OR**	**95% CI**	***p***	AOR^*^	**95% CI**	***p***
**Age (reference: 60–69 Years)**						<0.001			0.003			0.461
70–79	2.48	1.97–3.11	<0.001	1.96	1.48–2.60	<0.001	1.57	1.12–2.19	0.009	1.22	0.84–1.78	0.292
≥80	5.81	3.12–10.84	<0.001	2.47	1.17–5.22	0.018	2.74	1.37–5.48	0.004	1.49	0.68–3.28	0.324
Women (vs. men)	0.76	0.61–0.95	0.017	0.58	0.42–0.82	0.002	0.96	0.69–1.34	0.804	0.94	0.63–1.41	0.781
Not married (vs. married)	4.10	2.74–6.13	<0.001	3.58	2.15–5.97	<0.001	2.60	1.68–4.03	<0.001	2.50	1.19–3.53	0.009
Education (reference: primary)			<0.001			0.010			0.060			0.561
Middle	0.54	0.42–0.69	<0.001	0.63	0.46–0.87	0.004	0.74	0.52–1.05	0.088	0.94	0.63–1.41	0.772
High and above	1.11	0.70–1.75	0.654	0.96	0.52–1.74	0.880	1.31	0.72–2.38	0.318	1.33	0.68–2.60	0.412
Manual labour work (vs. office)	1.51	1.21–1.88	<0.001	1.32	1.00–1.74	0.053	1.71	1.23–2.41	0.002	1.42	0.98–2.05	0.064
Cohabiting (vs. alone)	0.69	0.52–0.90	0.007	0.84	0.58–1.23	0.377	0.93	0.62–1.40	0.730	1.11	0.67–1.84	0.687
Having children (vs. no)	0.33	0.19–0.57	<0.001	1.80	0.87–3.74	0.116	0.83	0.40–1.71	0.613	2.30	1.01–5.22	0.046
Currently Drinking (vs. no)	0.78	0.58–1.06	0.117	0.64	0.42–0.96	0.029	1.08	0.70–1.66	0.745	0.98	0.59–1.62	0.939
Currently Smoking (vs. no)	0.93	0.67–1.28	0.547	1.00	0.63–1.57	0.987	1.01	0.63–1.62	0.955	1.11	0.62–1.99	0.735
Without (vs. with) chronic conditions	0.52	0.41–0.64	<0.001	0.53	0.40–0.69	<0.001	0.56	0.40–0.78	0.001	0.65	0.45–0.92	0.015
Regular Exercise (vs. no)	1.00	0.80–1.25	0.995	1.26	0.94–1.69	0.120	0.65	0.47–0.90	0.010	0.67	0.47–0.97	0.032
Low (vs. high) disposable income	1.51	1.21–1.87	<0.001	2.21	1.54–3.18	<0.001	1.33	0.95–1.83	0.083	2.59	1.56–4.28	<0.001
Low (vs. high) satisfaction with income	2.66	2.13–3.32	<0.001	0.76	0.53–1.10	0.143	1.46	1.06–2.01	0.022	0.61	0.39–0.94	0.026
MCI (vs. Non–MCI)	13.33	9.67–18.41	<0.001	33.03	18.25–59.78	<0.001	3.63	2.61–5.51	<0.001	7.48	0.08–0.23	<0.001
Low income ^*^ MCI	7.03	4.74–10.43	<0.001	0.30	0.15–0.62	0.001	2.29	1.53–3.43	<0.001	0.32	0.15–0.64	0.002
R^2^				0.415						0.143	

*OR, Odds Ratio; AOR, Adjusted Odds Ratio; MCI, Mild Cognitive Impairment*.

* Predictors entered into the logistic regression models in a single step as a block, including age, gender, marriage, educational attainment, occupation, co-habiting arrangements, having children, currently drinking alcohol, currently smoking, chronic conditions, regular exercise, and satisfaction with disposable income, MCI, disposable income, and income^*^MCI.

**Figure 2:**
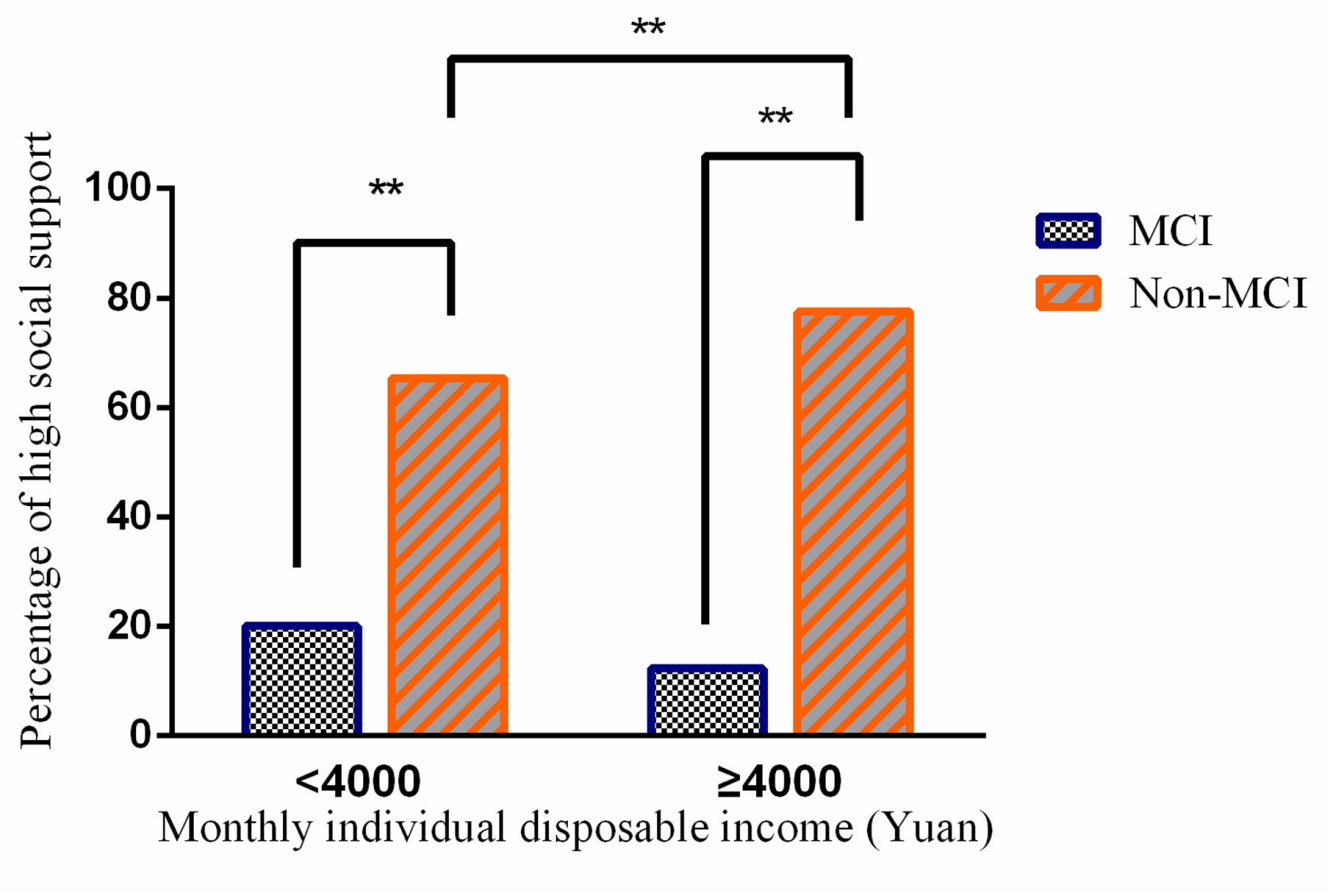
Gaps in social support between study participants with and without mild cognitive impairment (MCI) (**p < 0.001).

The gap in social support between those with and without MCI in the higher income group was larger compared with those in the lower income group (Figure 2). Further Chi-square tests showed that there was no significant income difference in the level of social support measured by DSSI in the study participants with MCI (*p* = 0.055), despite a significant higher level of DSSI in the higher income group without MCI compared with those in the lower income group (*p* < 0.001, significant with Bonferroni correction).

No significant additive interaction effects were found between MCI and disposable income on social support. The RERI, AP and S indicators were −2.978 (95% CI −9.599 to 3.643), −4.893 (95% CI −1.811 to 0.833), and 0.631 (95% CI 0.224 to 1.777), respectively, for the DSSI categories, and −12.183 (95% CI −37.145 to 12.778), −0.553 (95% CI −1.903 to 0.797), and 0.631 (95% CI 0.257 to 1.563), respectively, for the self-ratings on social support.

## Discussion

There is a lack of studies into the inequality in social support experienced by people living with MCI in China. We observed significant disparities in social support between those with and without MCI. The gap in social support between those with and without MCI is higher in the higher income group compared with those in the lower income group. High personal income is not linked with high social support in people living with MCI.

The study revealed lower social support, and lower satisfaction with social support, in the participants with MCI in comparison with their non-MCI counterparts. The results are consistent with findings of other studies (30, 62, 63). The relationship between MCI and social support is likely to be bi-directional. A meta-analysis of 43 longitudinal cohort studies showed that poor social relationships predict cognitive decline (30). Two principal theories were developed to explain this phenomenon. The mental stimulation theory proposes that “social support contributes to cognitive functions and neuronal growth by improving cognitive reserves through activation and strengthening of neurobiological pathways” (64). The stress-buffering theory posits that social support helps create a barrier to stressful reactions and subsequently reduces adverse psychological effects (65). In the other direction, some researchers believe that the decline of social support can be a consequence of MCI. Studies have shown that a self-perception of ageing and uselessness is associated with fewer social ties and lower frequency of social interactions (66). The decline of cognitive functions can deprive people of the willingness to socialise and lead to a loss of some critical skills that are deemed important for maintaining their existing social networks. The stigmatisation of MCI and dementia may also lead to social isolation, social rejection and internalised shame (67). A cross-cultural investigation across several countries including Italy, Poland and the United Kingdom revealed a negative association between stigmatisation and social support (22).

Socioeconomic inequalities in social support have been evident in a systematic review (68). This study adds further evidence to this literature. Low income is a significant predictor of low levels of social support, measured by both the DSSI scores and self-ratings. Income disparities in older people in Shanghai are unlikely to reduce any time soon as the vast majority receives a pension without any paid work. One of the interesting findings of this study is that self-ratings of social support decrease with high satisfaction with income, despite an absence of such an association when social support was measured by the DSSI scores. Similarly, lower self-ratings of social support were also found in those who had children compared with those who did not. Satisfaction with social support can be shaped by one's expectations. It is not clear whether those who had children and/or were satisfied with their income had adjusted their expectations of social support. Indeed, testing the impacts of different income levels on social support is complex, not least because of the difficulties in developing a comparable measurement (31). Some researchers have argued that the subjective experience of financial conditions is more important than the actual level of income or wealth as it is more closely related to health outcomes (69). However, our study does not seem to support such a claim. Unlike personal satisfaction with income, disposable income showed consistent associations with both the DSSI scores and self-ratings of social support.

The multiplicative interaction effect between income and MCI in associations with social support is concerning. High income brings benefits of high social support to those without MCI, but such benefits do not extend to those with MCI. This indicates that economic development and increased wealth will not solve the challenge of ensuring social support for people with MCI. It is important to note that although economic capital can be transformed into social capital and vice versa (27), the impacts of increased income on social support are limited. Our study indicates that high personal income can improve social support for people without MCI, but it plays a limited role, if any, for those with MCI.

Education has been understood to be one of the most important measures for reducing inequality (5). Good education enables people to develop essential communication and interpersonal skills required for taking advantage of social support. Education also improves economic participation and is often positively associated with income (27). In our study, however, those with middle school education reported the highest level of social support. Social support may even decline with further education. High levels of education do not always translate into more and stronger social ties. People with high levels of education may spend less time with their family due to commitments to sustained high levels of work obligations (27). In China, the “empty-nest” elderly with higher education reported higher levels of loneliness and social isolation compared with those with lower levels of education (70).

### Strengths and Limitations

This study addresses a gap in the literature by examining the interaction effect between MCI and income on social support. Social support was assessed using both objective and subjective measures. MCI was identified through a standardised two-phase protocol. The sample size was large.

This study has several limitations. Despite a large sample size, the study participants are not representative of the Shanghai population. In comparison with the older populations in Shanghai, the study sample was biased towards women and those aged between 70 and 79. Elderly people over 80 years were under-represented. Although the bias in the sample prevented us from generalising the results, it had limited implications for the testing of our research question: the association of social support with income and MCI. The population demographic profiles are likely to vary between residential communities and our study participants were recruited from two CHCs. The diversity in study samples across the two centres provides some advantages for this study in detecting variations of social support associated with the independent variables. We used multivariate regression analyses to control the confounding effects of covariates. Despite that, causal relationships should not be assumed due to the cross-sectional design.

Another limitation of the study is that most data were collected through a questionnaire survey. MCI may influence how the study participants perceived and responded to the questions, for example those embedded in the DSSI instrument. From the health services perspective, however, perceived social support is equally, if not more important, compared with the objective instrumental support in influencing healthcare seeking decisions, which is one of the key rationales behind this study.

It is also important to note that household income may impose a different effect on social support compared to individual income. Unfortunately, this study did not collect information about household income, although the potential confounding effects of co-habiting living arrangements and having children were controlled in the statistical analyses.

### Implications

Findings of this study have significant clinical practise and policy implications. Health care professionals have to consider both individual biopsychological characteristics and social circumstances of their patients in developing care plans for managing MCI. Low levels of social support can significantly jeopardise the accessibility and acceptability of MCI interventions (71). Unfortunately, people with MCI tend to experience lower social support than those without MCI. Policy makers and health care managers have the responsibility to foster an appropriate social environment to support the effective delivery of care for patients with MCI. High income is linked with high social support. However, income support alone is not enough to eradicate the disparity in social support between people with and without MCI. This study shows that the benefits of high income for social support do not extend to people with MCI. Given that the effective implementation of the currently available MCI intervention strategies depends heavily on social support, further studies into the underlying reasons for people with MCI having low social support are warranted. These studies should look at the problem from a broad social and cultural perspective, including but not limited to social stigma and ageism (2, 3). The lack of a link between high education and high social support also deserves increasing policy attention. Education should and can play a positive role in both social and economic development (69).

## Conclusion

In conclusion, there exist significant disparities in social support between people living with and without MCI in Shanghai, China. Such a gap is more profound in people with higher income. The inequality in social support associated with MCI may present a complex set of challenges to the successful implementation of community-based MCI detection and management programs.

## Data Availability Statement

The data analysed in this study is subject to the following licenses/restrictions: Data were provided by the Shanghai MCI study alliance, which are available upon reasonable request to the alliance through Jia Ma, Yalin Liu, and Dehua Yu. Requests to access these datasets should be directed to lyl56242@126.com.

## Ethics Statement

The studies involving human participants by Ethics approval was obtained from the Human Ethics Committee of La trobe University (HEC20125). Written informed consent for participation was not required for this study in accordance with the national legislation and the institutional requirements.

## Author Contributions

YLu contributed to the conceptualisation of the study, analysed and interpreted the data, and was the primary person responsible for drafting the manuscript. CL contributed to the conceptualisation of the study, guided data analyses and interpretation of the data, and critically revised the manuscript. SF critically revised the manuscript. JM, YLiu, and DY contributed to the design of the cross-sectional survey, data collection, and interpretation of the results of this paper. All authors read and approved the final manuscript.

## Funding

The project was supported by the fund from Shanghai Municipal Health Commission, Shanghai, China (201940495). This work was also supported by the Australian Government Research Training Program Fees Offset (RTP Fees Offset) and the La Trobe University Full Fee Research Scholarship (LTUFFRS). The funding bodies did not have any involvement in the design, execution, and writing up of the study.

## Conflict of Interest

The authors declare that the research was conducted in the absence of any commercial or financial relationships that could be construed as a potential conflict of interest.

## Publisher's Note

All claims expressed in this article are solely those of the authors and do not necessarily represent those of their affiliated organizations, or those of the publisher, the editors and the reviewers. Any product that may be evaluated in this article, or claim that may be made by its manufacturer, is not guaranteed or endorsed by the publisher.

## Abbreviations

MCI: mild cognitive impairment
CHCs: community health centres
DSSI: Duke Social Support Index
ISS: Instrumental Social Support
SIS: Social Interaction Scale
SSS: Subjective Social Support
RERI: relative excess risk due to interaction
AP: attributable proportion due to interaction
S: synergy index.
