# Supplementary material for: Inequality in Social Support Associated With Mild Cognitive Impairment: A Cross-Sectional Study of Older (≥60 Years) Residents in Shanghai, China
Source: Front Public Health. 2021 Nov 23;9:706322. doi: 10.3389/fpubh.2021.706322 (PMC8649958; doi:10.3389/fpubh.2021.706322)
Supplement: Supplementary file 1 [file Table_1.DOCX]

**Supplementary Table S1. Logistic regression results of factors associated with social support**

| **Predictor** | **DSSI score with a median split (1=Low; 0=High)** | | | | | |
| --- | --- | --- | --- | --- | --- | --- |
|  | **OR** | **95% CI** | ***p*** | **AOR*** | **95% CI** | ***p*** |
| **Age (reference: 60-69 Years)** |  |  |  |  |  | **<0.001** |
| 70-79 | 2.43 | 1.95-3.04 | **<0.001** | 1.92 | 1.46-2.54 | **<0.001** |
| ≥80 | 5.36 | 2.81-10.25 | **<0.001** | 2.01 | 0.92-4.38 | **0.080** |
| **Women (vs men)** | 0.81 | 0.65-1.01 | 0.065 | 0.65 | 0.46-0.91 | **0.011** |
| **Not married (vs married)** | 3.69 | 2.43-5.59 | **<0.001** | 2.74 | 1.62-4.62 | **<0.001** |
| **Education (reference: primary)** |  |  | **<0.001** |  |  | **0.001** |
| Middle | 0.50 | 0.38-0.62 | **<0.001** | 0.55 | 0.40-0.76 | **<0.001** |
| High and above | 0.99 | 0.63-1.58 | **<0.001** | 0.85 | 0.47-1.56 | 0.607 |
| **Manual labour work (vs office)** | 1.14 | 1.14-1.76 | **0.002** | 1.27 | 0.96-1.67 | 0.096 |
| **Cohabiting (vs alone)** | 0.60 | 0.45-0.79 | **<0.001** | 0.68 | 0.47-1.00 | **0.045** |
| **Having children (vs no)** | 0.34 | 0.19-0.60 | **<0.001** | 3.00 | 1.30-6.96 | **0.010** |
| **Currently Drinking (vs no)** | 0.72 | 0.54-0.98 | **0.033** | 0.61 | 0.41-0.91 | **0.017** |
| **Currently Smoking (vs no)** | 0.88 | 0.64-1.21 | 0.435 | 1.01 | 0.64-1.58 | 0.976 |
| **Without (vs with) chronic conditions** | 0.50 | 0.41-0.63 | **<0.001** | 0.49 | 0.37-0.64 | **0.010** |
| **Regular Exercise (vs no)** | 1.02 | 0.72-1.27 | 0.867 | 1.26 | 0.94-1.68 | 0.120 |
| **Low (vs high) disposable income** | 1.49 | 1.20-1.85 | **<0.001** | 1.85 | 1.30-2.63 | **0.001** |
| **Low (vs high) satisfaction with income** | 2.80 | 2.24-3.49 | **<0.001** | 0.84 | 0.58-1.20 | 0.338 |
| **MCI (vs Non-MCI)** | 19.87 | 13.36-29.54 | **<0.001** | 60.71 | 27.95-131.85 | **<0.001** |
| **Low income * MCI** | 9.73 | 6.07-15.59 | **<0.001** | 0.30 | 0.12-0.75 | **0.010** |

Note: AOR – Adjusted Odds Ratio; MCI – Mild Cognitive Impairment.
* Predictors entered into the logistic regression models in a single step as a block, including age, gender, marriage, educational attainment, occupation, co-habiting arrangements, having children, currently drinking alcohol, currently smoking, chronic conditions, regular exercise, and satisfaction with disposable income, MCI, disposable income, and income*MCI
